# Identifying sexual risk‐taking and ill health in the meeting with young people‐experiences of using an assessment tool

**DOI:** 10.1111/scs.13081

**Published:** 2022-04-21

**Authors:** Helena Kilander, Siw Alehagen, Sofia Hammarström, Marie Golsäter

**Affiliations:** ^1^ Jönköping Academy for Improvement of Health and Welfare, School of Health and Welfare Jönköping University Jönköping Sweden; ^2^ Department of Women’s and Children’s Health Karolinska Institutet Stockholm Sweden; ^3^ Department of Obstetrics and Gynaecology Eksjö Hospital Jönköping Sweden; ^4^ Linköping University Linköping Sweden; ^5^ Division of Nursing Sciences and Reproductive Health, Department of Health, Medicine and Caring Sciences Linköping University Linköping Sweden; ^6^ Region Västra Götaland Knowledge Centre for Sexual Health Gothenburg Sweden; ^7^ Division of Society and Health, Department of Health, Medicine and Caring Sciences Linköping University Linköping Sweden; ^8^ CHILD ‐ Research Group, School of Health and Welfare Jönköping University Jönköping Sweden; ^9^ Child Health Services Jönköping Sweden; ^10^ Department of Biomedical and Clinical Sciences Linköping University Linköping Sweden

**Keywords:** adolescent health, counselling, risk assessment, sexual identity, sexual orientation, sexual risk‐taking, victimisation, violence, youth

## Abstract

**Background:**

Identifying young people exposed to sexual risk‐taking or violence is fundamental, when seeking to strengthen their health. However, young people seldom share sexual health concerns or experiences of violence with healthcare professionals (HCPs). Studies evaluating how use of a risk assessment tool influences the dialogue about sexual health and violence are sparse.

**Aims:**

The aim of this study was to explore HCPs' experiences of using the SEXual health Identification Tool (SEXIT) in encounters with young people at Swedish youth clinics.

**Method:**

Three focus group interviews were conducted with 21 HCPs from nine youth clinics, where SEXIT had been introduced. Data were analysed using thematic content analysis.

**Results:**

Three themes were identified. The theme, *Facilitates dialogue about sexuality and vulnerability*, describes how the questionnaire pertaining to SEXIT helped to normalise and help both HCPs and young people to take part in the dialogue about sensitive issues. *Need for a trustful encounter* presents HCPs' ethical concerns regarding how the questionnaire affects the integrity of young people and trust‐making. *Sensitive topics entail challenges* describes HCPs' challenges when dealing with sensitive issues. Additionally, it describes needs for knowledge and collaboration when targeting vulnerable young people.

**Conclusions:**

The HCPs stated that using SEXIT developed their ability to address sensitive issues and helped both them and young people to take part in the dialogue about sexuality and exposure to violence. SEXIT involves experiences of ethical concerns regarding integrity and trust‐making. It also entails challenges in having dialogues about sensitive issues, how to deal with risk assessment outcomes and in improvements regarding inter‐professional collaborations.

## INTRODUCTION

Globally, early identification of sexual risk‐taking and ill health among young people defined as 10–24 years is fundamental, as this can affect their future health and relationships with others [1, 2, 3]. However, to identify young people exposed to or at risk of sexual ill health and to enable conversations is complex as this concerns sensitive topics involving cultural and religious aspects [1, 4, 5].

Furthermore, sexual risk‐taking and ill health are often associated with mental health issues and it can also include exposure to violence, which entails a need for inter‐professional collaboration [4, 6, 7]. It is therefore of great importance to explore how sexual health concerns or experiences of violence can be addressed, since young people seldom share these issues with HCPs on their own initiative [3].

Sexual risk‐taking is often described as risking unintended pregnancy [8] or ill health such as sexually transmitted infections (STI) [9, 10]. Factors associated with risk‐taking are for example sexual debut before the age of 15 [11], multiple sexual partners [10] and substance use [12]. Examples of young populations who are more exposed to violence and sexual risk‐taking are lesbian, gay, bisexual and transgender young people [13].

The phenomenon of sexual risk‐taking and ill health is an urgent issue in Sweden [14, 15, 16] as previous studies report a high prevalence of sexual violence in early adolescence among all genders [15], but especially among women [14, 16, 17] and transgender young people [16]. In a Swedish report, women (9.4%) reported experiences of a sexual crime significantly more often than men (1.4%) in 2019 [14]. There are also large differences between age groups. The proportion for both men and women is highest in the age group 20–24, where 31.6% of women report a sexual crim and 4.3% of the men [14]. About 13% of girls report experiences of penetrating sexual violence before 18 years of age [17].

In addition, male and transgender young people visiting youth clinics report more events of sexual risk‐taking such as multiple sexual partners [16], and there are also reports of increasing rates of gonorrhoea infections in the youth population [18]. Overall, this demonstrates a need for knowledge to understand how complex issues such as exposure to violence and experiences of multiple sex partners can be addressed and to identify young people in need of support and care.

In Sweden, there are outpatient care services called youth clinics, providing a wide range of services free of charge for young people 13–25 years of age, related primarily to sexual and psychosocial health concerns [19]. The majority of the visitors are women [19] and a recent study showed that 16% of young people had visited a youth clinic in the past 3 months [20].

SEXIT has been introduced in recent years at several youth clinics in Sweden [21]. The tool aims to facilitate the identification of young people exposed to or at risk of, sexual ill health and violence, and to promote a dialogue about these health concerns. SEXIT includes topics about sexual history, consumption of alcohol and drugs and experiences of violence. Previous small‐scale studies indicate that SEXIT is feasible and acceptable for use in clinical settings both by professionals and young people [21, 22]. Since these studies, SEXIT has been further developed to include more items, for example physical and emotional violence [21]. Studies are needed to increase knowledge about how use of SEXIT influences the dialogue about sexual health and violence. Our aim was to explore HCPs' experiences of using the SEXual health Identification Tool (SEXIT) in encounters with young people at Swedish youth clinics.

## METHODS

### Design

This was a qualitative study including focus group interviews (FGIs) [23]. The results are reported in accordance with the Consolidated Criteria for Reporting Qualitative Research (COREQ) checklist [24].

### Setting

In 2019, SEXIT was introduced to nine youth clinics in southeast Sweden, within a quality improvement collaboration between the youth clinics and the Knowledge Centre for Sexual Health in Region Västra Götaland. HCPs involved in the study were medical and psychosocial health professionals such as midwives, nurses, social workers and psychologists working at youth clinics.

### SEXIT

SEXIT is a tool developed specifically for youth clinics and is supposed to be used systematically with all visitors. It has three components: training for HCPs on how to use the tool, a questionnaire for young people and a handbook for HCPs. The handbook includes routines related to SEXIT, which responses indicate risks and suggestions for follow‐up questions and actions if there is a risk of ill health. An example of action is referral to psychologist for consultation. Visitors at youth clinics are offered to answer the questionnaire, followed by a HCP‐led dialogue with the young visitor about the responses in order to make a risk assessment [21].

### Sample and procedure

Youth clinics in both rural and urban settings were included, where HCPs probably meet young people from a variety of socioeconomic backgrounds and the access to youth clinics services differs. Visitors at youth clinics were offered to answer the questionnaire, followed by a HCP‐led dialogue with the youths about the responses in order to make a risk assessment [21].

In total, 45 HCPs, medical‐ and psychosocial health professionals, working at youth clinics where SEXIT were used, were invited to take part in the study. HCPs who had no clinical experience of using the tool were excluded.

Information about and an invitation to participate in the study were given by mail or orally at staff meetings. Out of 45 invited HCPs, two were excluded since they had no clinical experience and 22 declined to participate, while 21 decided to participate in the study. The median age of the HCPs was 50 years (range 28–63), 20 were women, 14 midwives or nurses and seven social workers. The HCPs had worked in their profession for 1–22 years (median 3.5 years). Regarding experience of using SEXIT, 14 of the HCPs had used it 6–20 times or more and seven HCPs 1–5 times.

An interview guide was developed within the research group, and feedback was given by two HCPs who did not participate in the study (Table 1). Three FGIs took place in meeting rooms at a conference facility during September 2019. Before starting the FGI, participants received information about the study once more and gave their written consent. The three FGIs had between six and eight participants and lasted 53–57 min. A moderator (HK) led the FGI and an assistant (MG) documented each session in writing. At the end of the session, the assistant presented a mind map of issues, which had been discussed in the current FGI and invited them to correct or comment upon the content. The FGIs were digitally recorded and transcribed verbatim afterwards. The transcribed data contained 43 pages of text.

**Table 1:** Interview guide for focus group interviews

Main questions (The questionnaire SEXIT was available at the table) 1. Tell me about your experiences when using the questionnaire SEXIT? ‐regarding different visits ‐Advantages ‐Challenges ‐ If you could change something, what would that be? 2. Tell me about your assessments in clinical practice when you have identified needs for support according to SEXIT? ‐Advantages ‐Challenges 3. How do medical health and psychosocial health care professionals collaborate at your clinic when you have identified needs for support, according to SEXIT? ***Final question*** Do You want to talk about something else that we haven’t mentioned?

### Analysis

A thematic analysis was used according to Braun and Clarke and followed six phases [25]. To become familiar with the data, the researchers read through each transcript and noted ideas and patterns. In the next phase, data extract, which corresponded to the aim were selected, and initial codes (*n* = 110) were generated and sorted. After that, the codes were re‐read and potential themes were collated. Thereafter, the 12 potential themes were reviewed by reading the collated extracts for each theme. The analysis involved searching for relationships between codes and themes, including recoding data. A thematic map was generated including all transcripts. The themes were then revised and illuminated to three main themes, which were named. Finally, a report was produced including the final analysis [25]. Table 2 illustrates an example of the analytic phases and Table 3 the subthemes and Main themes of the analysis. Three of the authors were responsible for the analysis process. Finally, the themes were discussed and agreed in the whole research group.

**Table 2:** Examples of the analysis

Data extract	Subtheme	Main theme
‘Even if I have asked questions about exposure to violence…I must be honest… I have not asked everybody previously…now everybody is asked the questions…’ FGI 3 ‘I consider if it is right timing in the visit …before I offer SEXIT*…*since it involves sensitive issues…’ FGI 2 ‘I don’t understand the topic… numbers of sexual partners during the last 12 months… is that relevant, why?’ FGI 1	Facilitates systematically questions about exposure to violence To establish trust requires time and presence in the meeting Challenges in applying a new approach and needs of knowledge	Facilitate dialogues about sexuality and vulnerability Need for a trustful encounter Sensitive topics entail challenges

**Table 3:** Subthemes and Main themes of the analysis

*Subthemes* Facilitates a systematically approach Facilitates systematically questions about exposure to violence Helps HCPs to normalise sensitive issues Promotes young people's participation in the conversation Opening up and creates focus in a conversation Needs of intuitive ability as questions may involve ethical dilemmas To establish trust requires time and presence in the meeting Limits trust‐making and the conversation Challenges in applying a new approach and needs of knowledge Uncertainty among HCPs when using the questionnaire in clinical practice Differences in collaborations Needs and requires improved inter‐professional collaboration	*Main themes* Facilitates dialogue about sexuality and vulnerability Need for a trustful encounter Sensitive topics entail challenges

In order to ensure that a good and reliable thematic analysis was provided the 15‐point ‘Checklist of criteria for good thematic analysis’ according to Braun & Clarke (25, page 96) was used as a guide throughout the analysis process. For example, all data were given equal attention during the coding process, and the analysis was a recursive process with movement back and forward.

Three authors have clinical experience of with meeting and counselling young people regarding sexuality. Two are midwives, and one is a paediatric nurse. The fourth author has a PhD and a master's degree in public health, specialised in sexual and reproductive health.

### Ethical considerations

The Swedish Ethical Board reviewed the study (# 2019/03628). HCPs gave their written informed consent to participate in the study.

## RESULTS

The analysis resulted in three themes: Facilitates dialogue about sexuality and vulnerability, Need for a trustful encounter and Sensitive topics entail challenges (Table 2). Quotes are shown to illustrate the themes.

### Facilitates dialogue about sexuality and vulnerability

The use of the questionnaire facilitated a more systematic approach in the visit with young people. HCPs described how it contributed to equal care by giving all young people the possibility to receive the same questions, and thereby it also helped to identify young people in need of support and care.

The HCPs stated that the questionnaire also helped them and young people to take part in the dialogue about sensitive issues and normalised topics such as gender identity, sexual orientation and exposure to violence. HCPs described how they previously often avoided these issues since they felt uncomfortable during the dialogue. They experienced that the questionnaire seemed to promote young people's participation through the possibility to first make a self‐assessment and reflect about one's own sexuality and health concerns before the conversation took place. Furthermore, HCPs stated that self‐assessment of alcohol or drug consumption contributed to more trustworthy responses compared with their previous use of open‐ended questions, asked orally.

The HCPs reported that the questionnaire also improved their ability to identify young people exposed to risk‐taking and violence, since it opened up the dialogue and created a focus about young people's living conditions. They stated that it also created a depth in the dialogue regarding individual extended needs for support or therapy that they probably would not have noticed otherwise.

*‘She had been raped one year ago… had declined support contact… but wanted it now… I wouldn't have known about the rape if I hadn't used SEXIT…’* (participant 1)

*‘I have not experienced that someone has reported about exposure to violence yet…but find it fair… that everyone gets the opportunity to speak out…’* (participant 2)

*‘…use of SEXIT has also facilitated reflections regarding violence among young people … a youth asked have I been exposed?’* (participant 3)

(FGI 1)

### Need for a trustful encounter

Offering the questionnaire resulted in a new situation which HCPs stated could challenge the integrity of young people and thus limit trust‐making. They wondered whether young people found the questionnaire valuable or felt uncomfortable when answering it, which affected their attitude towards using it.

The HCPs reported that a trustful encounter and intuitive ability is a condition for using the questionnaire. They stated that it required presence to establish a trustful relationship. Being present was described as being attentive, listening and following up on given answers. HCPs stated that with some young people, it could take several visits to establish trust before the young people were ready to answer the questionnaire and to talk about sensitive issues such as violence.

*‘It is central to be present in the conversation and to show you are interested in their lives…’* (participant 1)

*‘Being present… I agree … but I find it central …to dare to talk about complex issues… as in SEXIT… at the same time showing the young visitor that you will stay no matter what they answer’*. (participant 2)

(FGI 2)

An intuitive ability to decide when it is appropriate to offer and use the questionnaire seems fundamental to establish trust. For example, they described emotionally charged situations where the young visitor was so emotional that the HCPs postponed offering the questionnaire as they found it inappropriate.

Consultations with young people suffering from anxiety or stress were also complex. HCPs reported that the questionnaire could limit trust and the depth of the dialogue, since the questionnaire not involved mental health concerns. By using the questionnaire in those consultations, the HCPs stressed that young people's mental health concerns could be neglected. In contrast, HCPs also stated that from a holistic perspective, young people's mental and sexual health concerns are often interrelated.

### Sensitive topics entail challenges

HCPs in the youth clinics are used to discuss the topics of ill health and sexuality when meeting young people. The use of the questionnaire resulted in deeper aspects of sensitive issues and risk‐taking such as violence, sexual orientation, gender identity and number of sexual partners were investigated. They reported having limited knowledge and a need to understand why these sensitive issues were of importance when assessing sexual risk‐taking and ill health. They also described challenges in how to apply the questionnaire if young people had not initiated sex, and in meetings with young people not proficient in Swedish.

Ethical concerns regarding when and how the questionnaire should be applied in clinical practice were mentioned as a challenge. HCPs stressed questions about how to handle sensitive information from the questionnaire, for example how information should be registered in the medical record and shared within the team at the youth clinic or with social services. They also expressed uncertainty regarding responsibilities when dealing with sensitive issues such as violence in the questionnaire.

*‘He had forced someone else sexually…am I supposed to register this complex information in the record…it could be a crime…I don't know how to deal with that’*. (participant 1)

*‘I agree, that's one of the reasons why I feel unsecure about using SEXIT’*. (participant 2*)*

(FGI 3)

HCPs experienced that using the questionnaire both required and facilitated inter‐professional collaboration, especially when they were targeting vulnerable young people. Some youth clinics had daily inter‐professional meetings regarding young people in need of support, while others met occasionally. Lack of opportunities to collaborate affected how HCPs could respond to the outcome of the questionnaire. For example, HCPs at youth clinics who reported good opportunities for inter‐professional contact stated that this made it easier to transfer visitors between psychosocial and medical staff, depending on the assessment needs.

## DISCUSSION

This study highlights how the use of SEXIT enhanced HCPs' capability to address issues and *facilitated dialogue about sexuality and exposure to vulnerability*. The findings reflect HCPs' experiences of ethical concerns when using SEXIT and the *need for a trustful encounter* as well as how deeper aspects of *sensitive topics entail* need for new skills when dealing with outcomes.

HCPs experienced that SEXIT facilitated dialogue about sexuality and vulnerability. To our knowledge, few studies have reported how a questionnaire can help HCPs when having dialogues with young people about both risk‐taking and violence. Previous studies confirm the feasibility and acceptance of using a questionnaire when seeking to identify sexual risk‐taking [21, 22, 26] as well as the effectiveness of use of a questionnaire in distinguishing exposure to violence [4]. Exposure to physical, emotional and sexual violence has wide negative health effects [3, 27, 28]. By helping young people to disclosure experiences of violence and by offering support or treatment, young people's health could ultimately be strengthened and subsequent violence could be prevented.

Our results also describe how both HCPs and young people seemed to participate in the dialogue about risk‐taking to a greater extent. HCPs experienced that SEXIT facilitated the dialogue about sexuality and vulnerability because not only it provided support for them, but also the young people got an opportunity to reflect about their own sexuality and health concerns, before the conversation. In a wider perspective, the use of SEXIT could strengthen young people's autonomy in conversations about sexual health concerns, as young people have the opportunity to take an equal part in the conversation. Previous research highlights that active participation of adolescent survivors of commercial sexual exploitation in consultation with health care, helps them to reclaim their autonomy over decisions affecting their health, like caring about themselves and taking control over their bodies [29]. Further research is needed from the perspective of young people, especially since also sexual scripts and norms regarding sensitive topics seem to differ between HCPs and young people.

HCPs stated a need for a trustful encounter. They experienced that some topics such as the number of sexual partners could inhibit trust, and stated the importance of having an intuitive ability to understand when it was appropriate to offer SEXIT. In contrast, young people seem to appreciate when HCPs address issues regarding SRHR, valuing it as important and not uncomfortable to talk about [21]. HPCs' focus on trust‐making in our study before addressing sensitive topics, such as sexuality, could be explained by the fact that young people seem to have a more liberal view than the HCPs regarding sexuality, and an openness to talk about it [19, 20]. The cautious approach regarding violence among the HCPs can also explain why it can take several visits until young people are asked questions about exposure to violence [3].

The HCPs in our study experienced that sensitive topics entail challenges, this highlights a need for more knowledge and skills among HCPs in having dialogues and dealing with information concerning sexual identity, orientation and violence. This is especially true for lesbian, gay, bisexual [4] and transgender young people since they are more exposed to violence and sexual risk‐taking [13] and may experience more difficulties in disclosing risk‐taking and exposure to violence due to a general stigma in the society. Increased knowledge and training in the model of addressing sexual functioning (PLISSIT model) could help HCPs with feeling comfortable on how to introduce SEXIT during the encounter and raise sexual issues reported in SEXIT [30]. The open‐ended questions in the PLISSIT model might also facilitate the conversation about violence and sexual risk‐taking reported in SEXIT [30].

Our study reflects a need for better articulated clinical routines. For example, HCPs described a lack of knowledge on how to deal with risk assessment outcomes and a need for improving access to equal care at youth clinics in rural settings. Additionally, we found a need for enhancing collaboration with social welfare services regarding vulnerable groups of young people in rural settings, confirming the findings of similar studies [20].

## METHODOLOGICAL CONSIDERATIONS

A strength of this study is that the participants represented different professionals and youth clinics from both rural and urban settings. Furthermore, the number of participants in the FGIs is in accordance with the literature [23]. There was also a variety in professional experience and a diversity of ages among the HCPs, which strengthens the credibility of the study and increases the likelihood that our findings will be more widely transferable to other youth clinics in Sweden using SEXIT [31].

A limitation of the study was that some of the participants had only used SEXIT one to five times. Their experiences could differ from those with greater experience of using SEXIT. Another limitation is that few men were included, although this mirrors the gender distribution among HCPs working at youth clinics in Sweden.

Dependability was considered by presenting the context and the approach in the method section. Confirmability was strengthened by describing the phases in the analysis and linking the findings to the transcripts by presenting data extracts [31].

## CONCLUSIONS

Using SEXIT improves HCPs ability to have dialogues concerning sexual risk‐taking and exposure to violence. The HCPs state that it also helps both HCPs and young people to take part in the conversation. Additionally, HCPs describe how SEXIT involves ethical concerns regarding young people's integrity, and how usage may affect trust‐making. HCPs need more knowledge and skills regarding having dialogues about infrequently discussed sensitive issues such as sexual identity, sexual orientation and violence. Furthermore, organisational challenges exist regarding how to deal with risk assessment outcomes and how to improve inter‐professional collaboration.

## CONFLICT OF INTEREST

The authors report no conflicts of interest in connection with this manuscript.
